# Use of Multiple Bacteriophage-Based Structural Color Sensors to Improve Accuracy for Discrimination of Geographical Origins of Agricultural Products

**DOI:** 10.3390/s21030986

**Published:** 2021-02-02

**Authors:** Daun Seol, Daeil Jang, Kyungjoon Cha, Jin-Woo Oh, Hoeil Chung

**Affiliations:** 1 Department of Chemistry and Research Institute for Convergence of Basic Science, Hanyang University, Seoul 04763, Korea; du_90@naver.com; 2 Department of Mathematics and Research Institute for Convergence of Basic Science, Hanyang University, Seoul 04763, Korea; paranhanel88@naver.com (D.J.); kjcha@hanyang.ac.kr (K.C.); 3 Department of Nanoenergy Engineering, Pusan National University, Busan 46241, Korea

**Keywords:** bacteriophage-based structural color sensor, multiple color sensors, discrimination of geographical origin, garlic, onion, perilla

## Abstract

A single M13 bacteriophage color sensor was previously utilized for discriminating the geographical origins of agricultural products (garlic, onion, and perilla). The resulting discrimination accuracy was acceptable, ranging from 88.6% to 94.0%. To improve the accuracy further, the use of three separate M13 bacteriophage color sensors containing different amino acid residues providing unique individual color changes (Wild sensor: glutamic acid (E)-glycine (G)-aspartic acid (D), WHW sensor: tryptophan (W)-histidine (H)-tryptophan (W), 4E sensor: four repeating glutamic acids (E)) was proposed. This study was driven by the possibility of enhancing sample discrimination by combining mutually characteristic and complimentary RGB signals obtained from each color sensor, which resulted from dissimilar interactions of sample odors with the employed color sensors. When each color sensor was used individually, the discrimination accuracy based on support vector machine (SVM) ranged from 91.8–94.0%, 88.6–90.3%, and 89.8–92.1% for garlic, onion, and perilla samples, respectively. Accuracy improved to 98.0%, 97.5%, and 97.1%, respectively, by integrating all of the RGB signals acquired from the three color sensors. Therefore, the proposed strategy was effective for improving sample discriminability. To further examine the dissimilar responses of each color sensor to odor molecules, typical odor components in the samples (allyl disulfide, allyl methyl disulfide, and perillaldehyde) were measured using each color sensor, and differences in RGB signals were analyzed.

## 1. Introduction

Correct identification of the geographical origin of agricultural products is an essential step to fair evaluation of their commercial value. Separation methods, such as LC-MS [1,2,3], which are able to analyze the components of each sample, are typically adopted for this purpose. Due to the rapid increase in international trading of agricultural products, large numbers of samples originating from different countries must be analyzed. Therefore, a simple and rapid screening method enabling on-site measurement is needed. To address this demand, our research group previously demonstrated the M13 bacteriophage-based color sensor (hereinafter, simply referred to as color sensor), which exhibits structural color change on interaction with gaseous molecules to discriminate the geographical origins of three different agricultural products (garlic, onion, and perilla) [4]. The swelling of self-assembled phages on the color sensor induced by interaction of its amino acid residues with odor components changes its structural color, and the dissimilar composition of odors in each sample enabled discrimination of geographical origin. Although the feasibility of this methodology was previously found, further improvement of accuracy such as by enhancing the discriminability of odor components was necessary to facilitate practical use of the color sensor.

Since the physical interaction of target odors with amino acids on the sensor’s surface is the source of the color change, the phage-based color sensor’s responses are not highly chemical structure-specific. Thus, it is difficult to differentiate components with similar molecular structures, and the selectivity of a given color sensor is not high. To overcome this limitation, a strategy utilizing multiple color sensors, wherein the surface of each color sensor possesses different amino acid residues with diverse functional groups and integration of these complimentary RGB signals for discriminant analysis, was explored in this study. With this approach, individual components in an odor are more discriminable since the interactions of a component with each color sensor are dissimilar, and subsequent color changes are characteristic. Combined color changes could be more component-descriptive and advantageous for improving the discrimination of samples. In parallel, the introduction of different amino acid residues into the M13 bacteriophage can be readily accomplishable by genetic engineering [5,6,7,8,9,10].

To examine the accuracy of the proposed approach, three multiple bacteriophage-based color sensors containing different amino acid residues (Wild sensor: glutamic acid (E)-glycine (G)-aspartic acid (D), WHW sensor: tryptophan (W)-histidine (H)-tryptophan (W), 4E sensor: four repeating glutamic acids (E)) were prepared. The Wild sensor contained hydrophilic (glutamic and aspartic acid) and hydrophobic (glycine) components, and the WHW sensor [11] consisted of hydrophilic histidine and an amphipathic tryptophan, which contains an indole ring. The surface of the 4E sensor is the most hydrophilic due to its four consecutive glutamic acid units [12,13]. Therefore, the surface characteristics of the color sensors differed. RGB signals of the odors from three agricultural products (garlic, onion, and perilla) were acquired using the color sensors, and the color changes that occurred with each color sensor were compared. Next, RGB signals from each color sensor were used separately to discriminate the geographical origin of the samples using support vector machine (SVM) [14,15,16]. In addition, RGB signals obtained from each color sensor were combined, and these integrated RGB data were used for discrimination. Discrimination accuracy using single and multiple color sensors was compared. In addition, for detailed investigation of color sensor response to odor-generating components, odor molecules (allyl disulfide, allyl methyl disulfide, and perillaldehyde) common to the employed agricultural samples were individually measured, and their RGB signals were analyzed in relation to their molecular structure and the respective amino acid residues on the sensors.

## 2. Materials and Methods

### 2.1. Sample Preparation and Measurement of Samples Using Color Sensors

**Figure 1:**
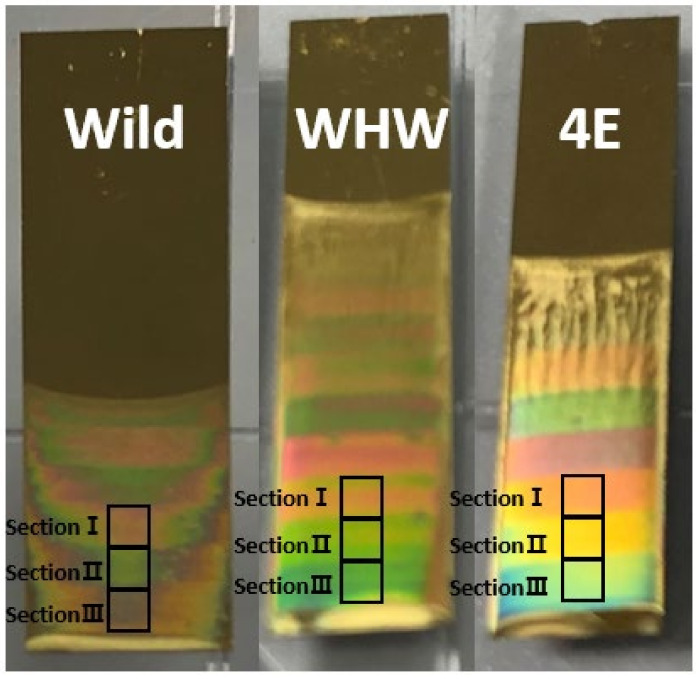
Wild, WHW, and 4E sensors containing the amino acid residues of glutamic acid (E)-glycine (G)-aspartic acid (D), tryptophan (W)-histidine (H)-tryptophan (W), and four repeating glutamic acids (E), respectively.

The same garlic, onion, and perilla samples used in our previous publication [4] were employed in this study. For each product, a total of 40 samples (20 domestic and 20 imported samples) harvested in various regions were collected to incorporate a wide compositional variation in the sample set, as previously described. Initially, M13 bacteriophages were prepared in a Tris-buffered saline solution and allowed to self-assemble on an Au-coated Si wafer via a pulling technique [9,17,18]. The concentration of M13 bacteriophages in the buffer solution was 5 mg/mL. Three sections (referred to as sections I, II, and III) of self-assembled bacteriophages were produced on the sensor surface by varying the pulling speed. Figure 1 shows the Wild, WHW, and 4E sensors and sections I, II, and III in each sensor. The colors on the sensor are from the reflection of white LED light.

**Figure 2:**
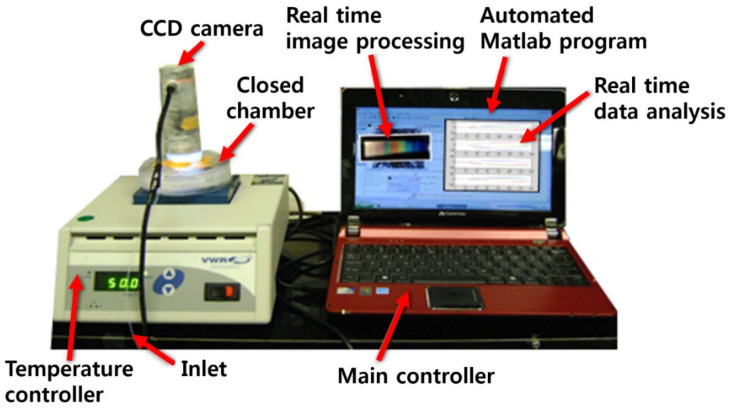
Overall experimental setup for measurement of samples using color sensors.

A razor-cut 1.0-g piece of garlic, a 1.0-g piece of onion, and 0.2 g of perilla seeds were prepared for color sensor analysis. Each prepared sample was introduced into a 500-mL sealed plastic chamber containing color sensors for measurement. After loading a piece of sample on a metal heating block, the temperature was increased from 30 °C to 60 °C in 40 s (0.75 °C/s) and was maintained at 60 °C for 252 s. Then, the temperature was again elevated from 60 °C to 90 °C over 40 s and was kept at 90 °C for 252 s. During the temperature elevation, RGB signals were acquired on sections I, II, and III of the color sensor using a CCD microscope camera (Celestron LCC., Torrance, CA, USA). Figure 2 shows the overall experimental setup including the closed measurement chamber embedded with a CCD camera, temperature control unit, and data acquisition/processing unit. Each color sensor was positioned under the CCD camera. 

### 2.2. Discriminant Analysis Using SVM

For the SVM kernel function, linear as well as radial basis functions [19,20] were evaluated. In cases employing a linear kernel, the degree varied from 1 to 5, and simultaneously the cost constant (C) changed from 1, 10, 100, 1000, to 10,000. Next, the optimal combination of the degree and cost constant yielding maximized discrimination performance was identified. When the radial basis function was adopted, the sigma varied from 1, 10, 100, 1000, to 10,000 with the same variation of cost constant to assess discrimination accuracy at different combinations. To run the SVM, R 3.2.0 software (R Development Core Team., 2005) provided by the Comprehensive R Archive Network (CRAN) at http://cran.r-project.org/ was employed.

## 3. Results

### 3.1. Examination of RGB Signals of Garlic Samples Measured with Each Color Sensor

**Figure 3:**
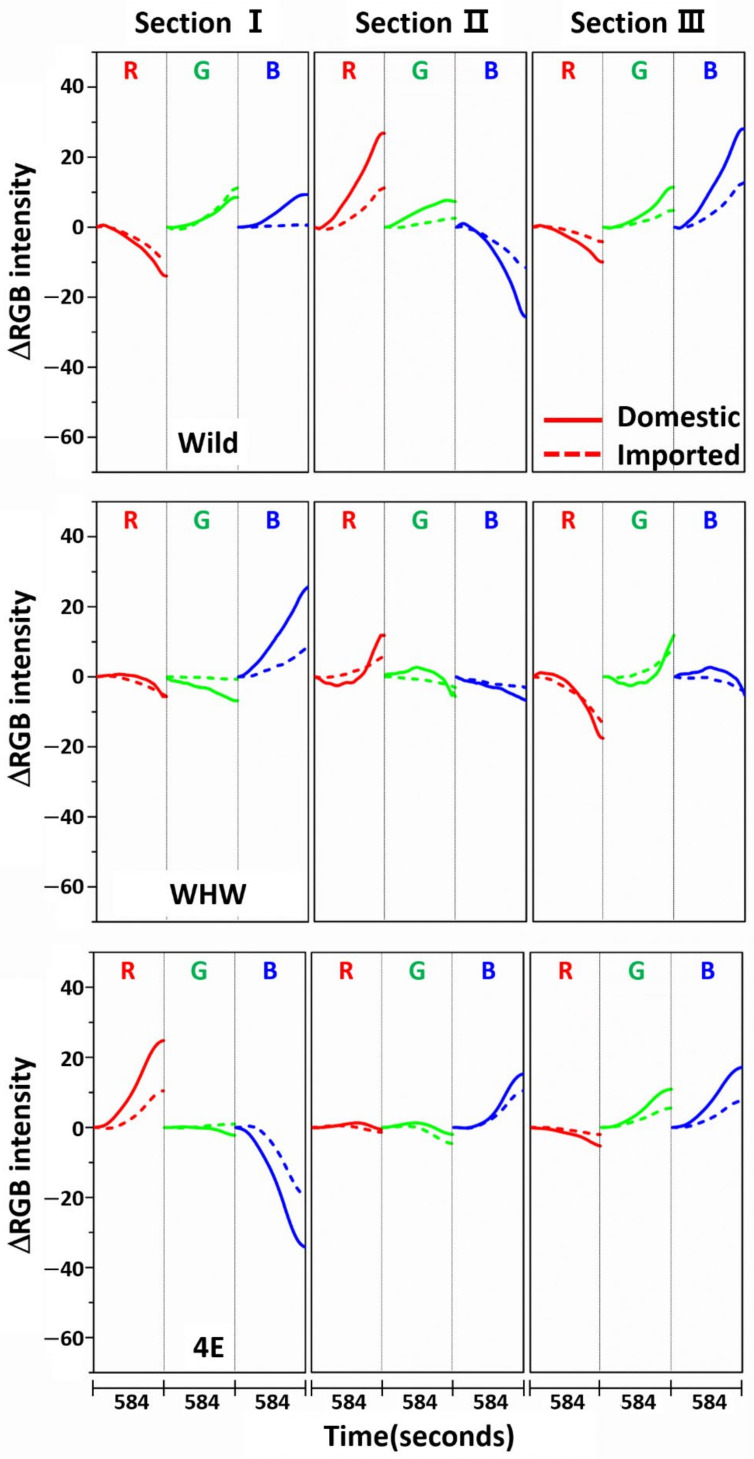
Average ΔRGB intensity of garlic samples measured in the three sections of Wild, WHW, and 4E sensors. The solid and dotted lines indicate the domestic and imported samples, respectively.

**Figure 4:**
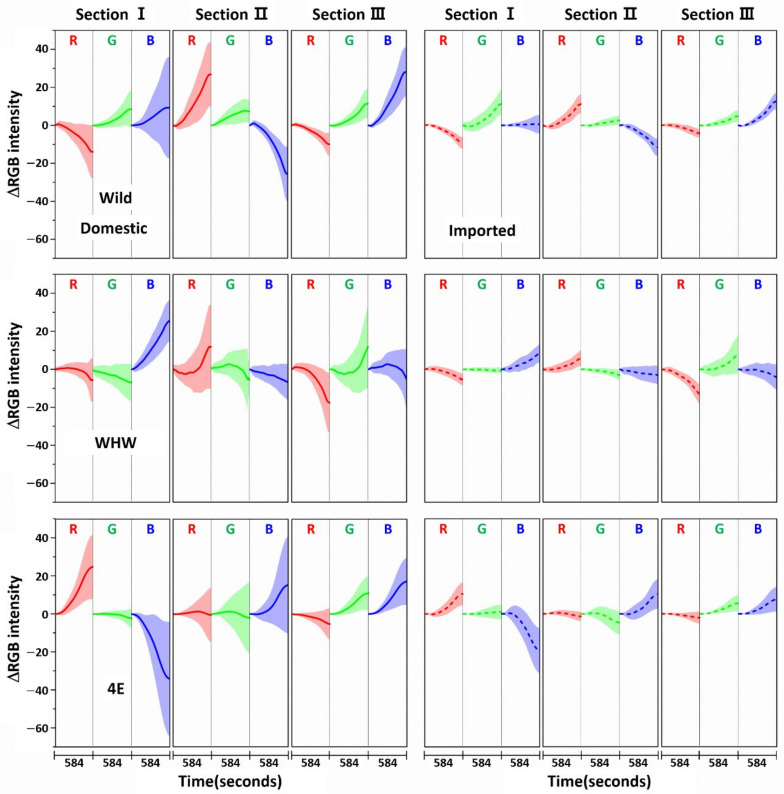
The same ΔRGB intensities shown in Figure 3 with standard deviation (shading) for the domestic (left plot) and imported samples (right plot).

Figure 3 shows the variation in average ΔRGB intensity at the three sections of the Wild (first row), WHW (second row), and 4E (third row) sensors in the measurement of garlic samples. The ΔRGB intensity corresponded to the change in color relative to the control in the absence of sample. Domestic and imported garlic samples are represented by solid and dotted lines, respectively. For each case, the average of 60 ΔRGB intensities obtained from 20 samples (triplicate measurements for each sample) is shown. The same ΔRGB intensities with standard deviation (indicated by the overlapped shades) for the domestic (left plot) and imported garlic samples (right plot) are presented in Figure 4. The standard deviation represents the intensity variation among the samples. The variation in RGB signals, such as direction and magnitude of color change, in the measurements using Wild and WHW sensors is generally analogous, except that blue color changes differed considerably.

The observed color changes originated from two causes. First, upon adsorption of odor components on the sensor, M-13 bacteriophage bundles swelled and/or shrunk in response, resulting in changes in the coherent scattering from the phage bundles, subsequently promoting distinct structural color change [6]. Second, the level of HOMO-LUMO in amino acid residues on the sensor surface shifted after adsorption of odor components, which changed the refractive index of the amino acid residues [21]. The varied refractive index caused the observed color changes. As described earlier, hydrophilic (glutamic and aspartic acid) and hydrophobic (glycine) components were both present in the Wild sensor, and the WHW sensor consisted of a hydrophilic histidine and amphipathic tryptophan, which contained a benzene ring. Therefore, the surface of the WHW sensor was slightly more hydrophilic than that of the Wild sensor, but not noticeably different, which explains the analogous color changes for the two sensors. Meanwhile, since the aromatic ring in the WHW sensor was able to induce π-π interaction, the interactions of the color sensors with the garlic odor components were expected to be somewhat dissimilar, as seen with the variation in blue color change.

**Figure 5:**
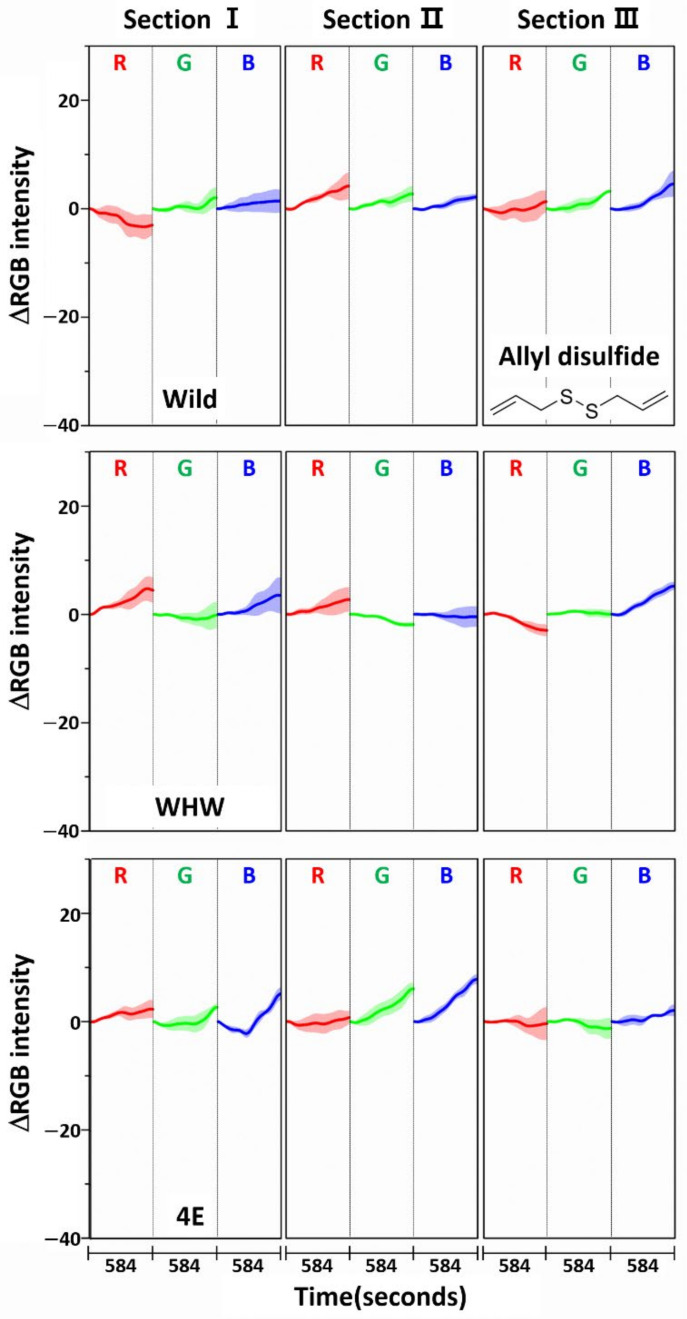
Average ΔRGB intensity of allyl disulfide acquired using Wild, WHW, and 4E sensors. The molecular structure of allyl disulfide is also shown.

The color change patterns acquired using the 4E sensor were considerably different from those using the Wild and WHW sensors. In particular, the directions of red and blue color changes in sections I and II were opposite. The surface of the 4E sensor was composed of four consecutive glutamic acid units and was mostly hydrophilic; its interaction with the odor components was obviously dissimilar to the other color sensors. Overall, the variation in RGB signals obtained from the three color sensors was individually characteristic due to the differences in surface polarity and functional groups. The magnitude of standard deviation (shaded areas) was greater among domestic samples, which indicated that the odor composition in the domestic samples was more diverse and/or the concentration of odor components was higher. According to a previous report, sulfur-containing compounds such as methyl methylthiomethyl disulfide and di-(thiomethylmethyl) disulfide are the predominant components (comprising more than 60~65%) of the approximately 50 volatile components including monoterpenes present in garlic odor [22]. Thus, it was of interest to examine the responses of each color sensor when a single odor component was measured. Allyl disulfide was chosen as a representative odor compound and was measured using the color sensors under the same experimental conditions. The results are shown in Figure 5. The average ΔRGB intensities based on three replicate measurements and the corresponding standard deviation (shaded areas) are also presented. As expected, the intensities were much smaller (note the magnitude of the *y*-scale) compared to those obtained from the garlic samples, because the RGB signals resulted from only a single component rather than multiple components. In addition, the color change patterns were dissimilar from sensor to sensor; in particular, the blue color change for the 4E sensor was more distinct from that of the Wild and WHW sensors. Similar to the measurement of garlic samples, the 4E sensor exhibited more different variation in color change in the measurement of the single allyl disulfide component.

### 3.2. Examination of RGB Signals of Onion Samples Measured with Each Color Sensor

**Figure 6:**
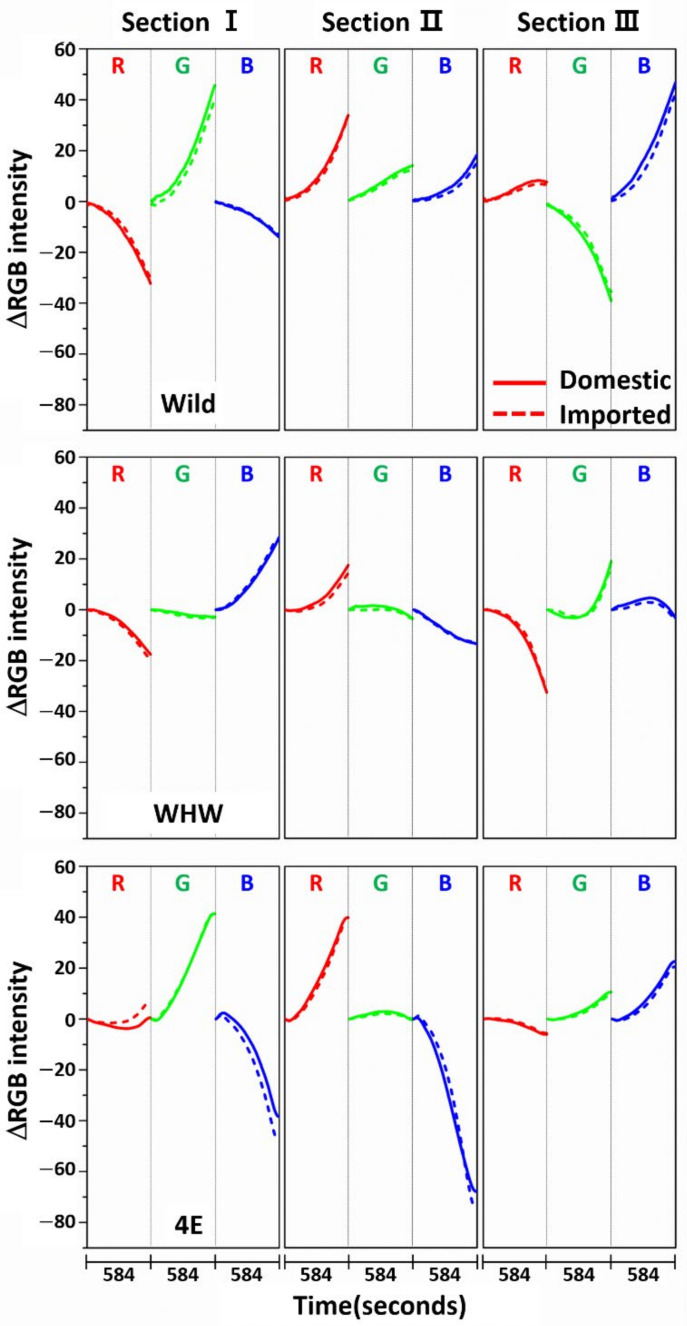
Variation of average ΔRGB intensity occurring in the three sections of Wild, WHW, and 4E sensors in the measurement of onion samples (solid line: domestic sample, dotted line: imported sample).

**Figure 7:**
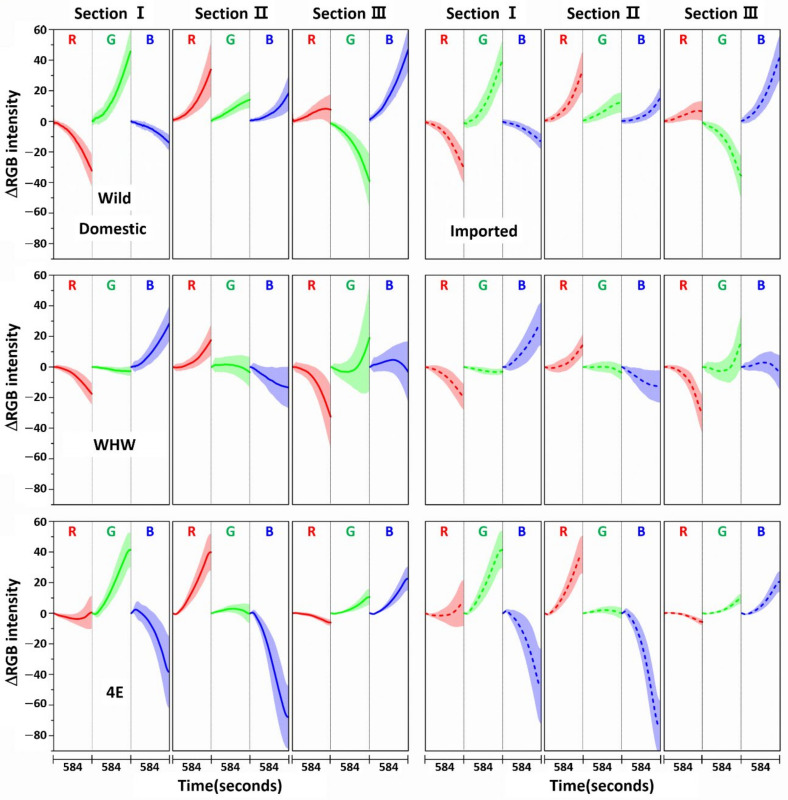
The same ΔRGB intensities shown in Figure 6 with standard deviation (shading) for the domestic (left plot) and imported samples (right plot).

Figure 6 shows variation in average ΔRGB intensity that occurred in the three sections of the Wild (first row), WHW (second row), and 4E (third row) color sensors in the measurement of onion samples (solid line: domestic sample, dotted line: imported sample). Figure 7 indicates the same average ΔRGB intensities for the domestic (left plot) and imported garlic samples (right plot) with standard deviation. The most noticeable observation is that the overall color change magnitudes were larger (note the magnitude of the *y*-scale) than those of garlic samples. In particular, the blue color changes in sections I and II of the 4E sensor were considerably larger, and their directions were reversed. The overall large color changes probably originated from the strong interaction of pungent odor components with the color sensors and/or a large amount of odor components.

**Figure 8:**
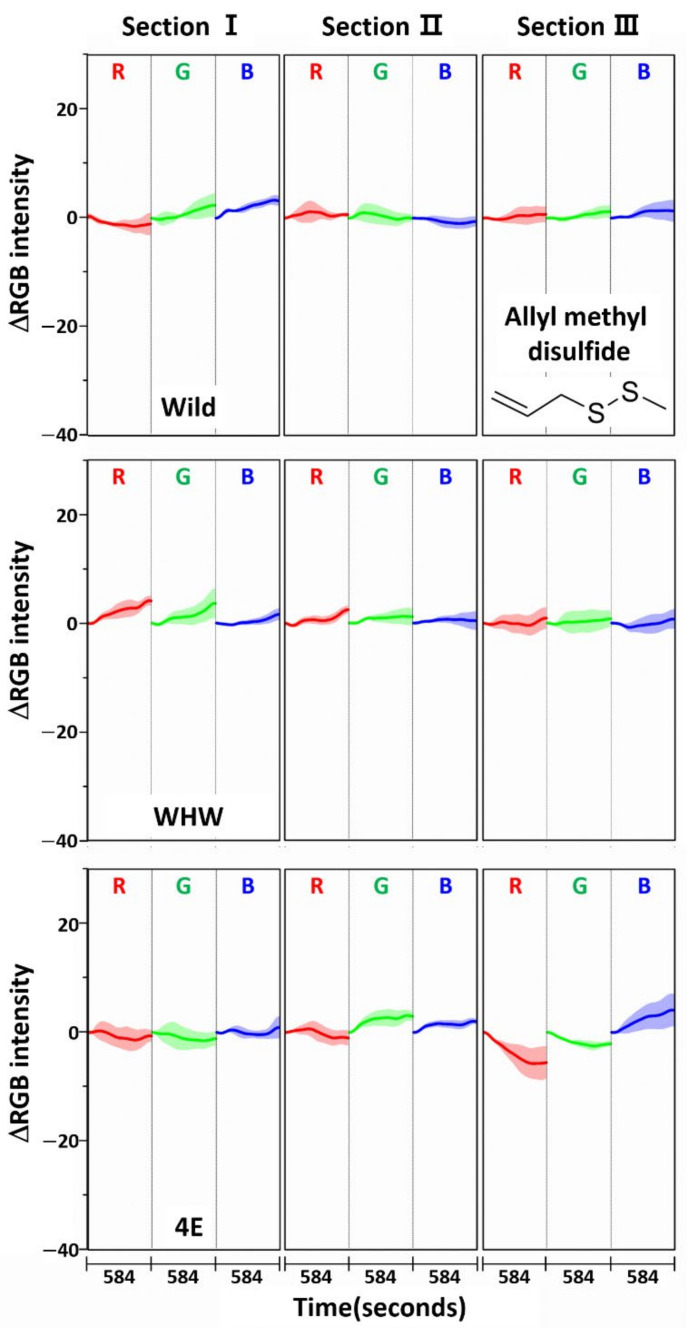
Average ΔRGB intensity of allyl methyl disulfide individually acquired using Wild, WHW, and 4E sensors. The molecular structure of allyl methyl disulfide is also shown.

The RGB change patterns observed from the color sensors were mutually different due to the interactions of onion odor components with the dissimilar amino acid residues on each sensor. The concentration of sulfide species responsible for the intense onion smell comprised nearly 90% of the total odor components, with dipropyl disulfide being the most abundant (53%), as noted in a previous paper [23]. Therefore, allyl methyl disulfide, which has a similar molecular structure to dipropyl disulfide, was chosen for single component analysis, and its RGB signals on each color sensor examined, as shown in Figure 8. Again, the color change magnitude was small due to exposure to only a single component. The color change patterns obtained from the Wild and WHW sensors were slightly different from each other. Of note, there was a large change in section III of the 4E sensor with different patterns, suggesting a stronger interaction of allyl methyl disulfide with glutamic acid residues.

### 3.3. Examination of RGB Signals of Perilla Samples Measured with Each Color Sensor

**Figure 9:**
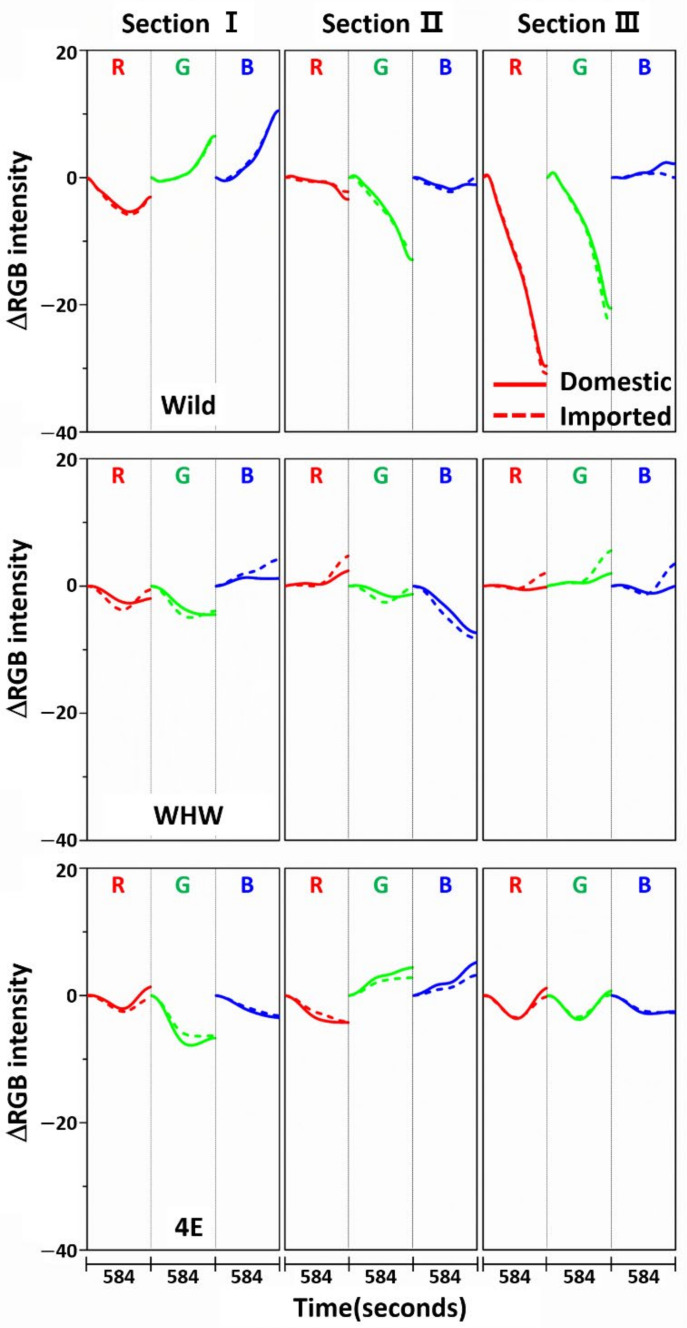
Average ΔRGB intensity of perilla samples measured in the three sections of Wild, WHW, and 4E sensors. The solid and dotted lines indicate the domestic and imported samples, respectively.

**Figure 10:**
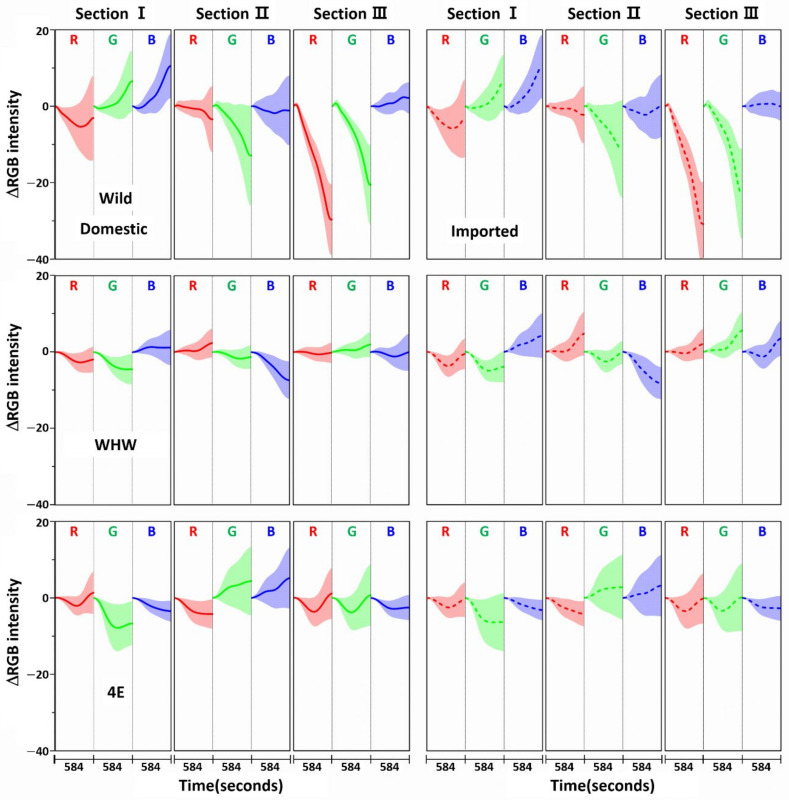
The same ΔRGB intensities shown in Figure 9 with standard deviation (shading) for the domestic (left plot) and imported samples (right plot).

Figure 9 shows the variation in average ΔRGB intensity in the three sections of the Wild (first row), WHW (second row), and 4E (third row) sensors in the measurement of perilla samples (solid line: domestic sample, dotted line: imported sample). Figure 10 shows the same average ΔRGB intensity with standard deviation for the domestic (left plot) and imported perilla samples (right plot). Distinctly different from the measurement of garlic and onion samples, the overall color change magnitudes were much smaller (note the magnitude of the *y*-scale), which indicates much weaker interactions of perilla odor components with the color sensors. It was previously found that 1-(3-furanyl)-4-methyl-1-pentanone (perillaketone) was the major component (44.4–69.2%) in perilla odor, followed by isoegomaketone (7.3–27.6%), and 16 other components [24].

**Figure 11:**
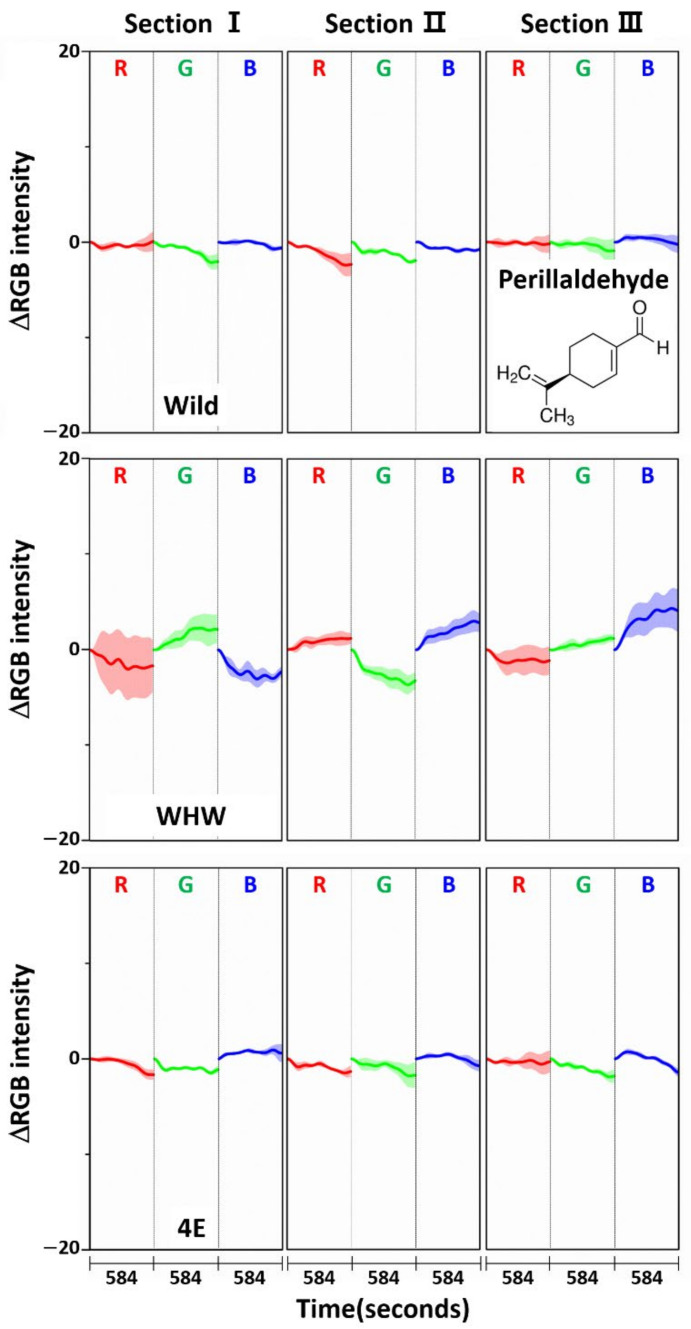
Average ΔRGB intensity of perillaldehyde individually acquired using Wild, WHW, and 4E sensors. The molecular structure of perillaldehyde is also shown.

Since these components are relatively hydrophobic, their interactions with the color sensors were weak; therefore, the overall observed color change magnitudes were small. Another interesting observation was that the color variations acquired by the Wild sensor were greatest, especially red and green colors on section III, since the surface of the Wild sensor is relatively more hydrophobic compared to the other sensors. Again, the color change patterns observed from all the color sensors were somewhat different, and the obtained RGB signals were mutually complimentary for sample discrimination. To examine RGB responses for a single component, perillaldehyde (a major component in perilla oil) [25,26,27] was chosen since perillaketone and isoegomaketone were difficult to obtain. Figure 11 shows the ΔRGB intensity of perillaldehyde acquired using each color sensor. In comparison with the measurements of allyl disulfide (Figure 5) and allyl methyl disulfide (Figure 8), the intensity variation measured with the Wild and 4E sensors was considerably smaller. This confirms weak interaction of perillaldehyde with these two color sensors. Meanwhile, the color changes were more notable on the WHW sensor, which possessed an aromatic residue. The increased variation probably originated from π-π interactions between the double bond in perillaldehyde and the indole in the WHW sensor.

### 3.4. Examination of Discrimination Accuracy Using SVM

**Figure 12:**
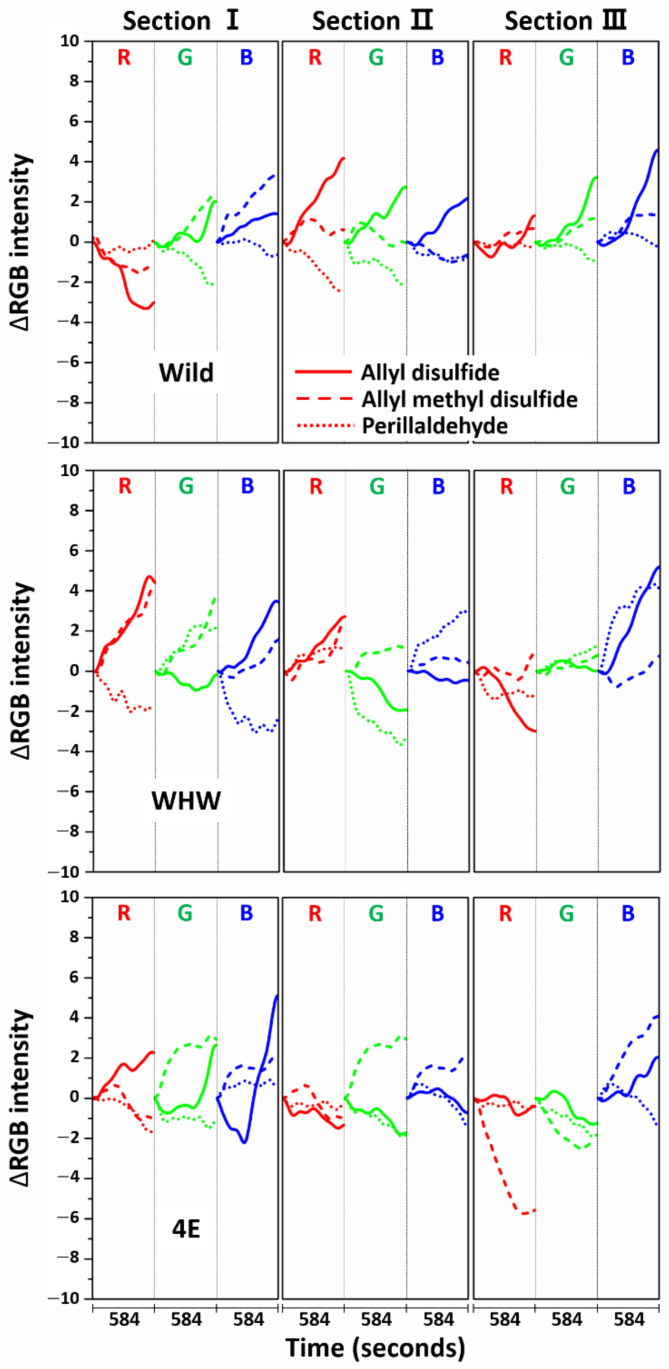
Average ΔRGB intensity of allyl disulfide, allyl methyl disulfide, and perillaldehyde measured using Wild (top), WHW (bottom), and 4E sensors (bottom).

Before the estimation of discrimination accuracy, the specificity of each sensor was examined by comparing the RGB signals measured from allyl disulfide, allyl methyl disulfide, and perillaldehyde, as shown in Figure 12. The allyl disulfide and allyl methyl disulfide signals are of particular interest, since the molecular structures of both components are similar except the number of double bonds. The RGB signal shapes of allyl disulfide and allyl methyl disulfide at each section of the color sensors varied, although the red color signals were relatively similar in sections I and II of the WHW sensor. This demonstrates that the responses of each color sensor were molecular structure-dependent and able to differentiate odor molecules with analogous structure. In the case of perillaldehyde, the RGB signals were generally dissimilar to those of allyl disulfide and allyl methyl disulfide, except the blue signal in section II of the Wild sensor, the red signal in section III of the Wild sensor, the green signal in section I of the WHW sensor, the green signal in section III of the WHW sensor, and the red signal in section II of the 4E sensor. Overall, each color sensor responded differently to the odor molecules and the sensor-to-sensor responses were independent. 

The RGB data measured from the three sections on each color sensor were adopted for the discrimination of domestic and imported samples using SVM. The accuracy was evaluated with 1000-time cross validation by randomly selecting 15 samples from each group for training and employing the remaining five samples for validation. The obtained accuracy, sensitivity, and specificity calculated by ((TP + TN)/(TP + FP + FN + TN)) × 100, (TP/(TP + FN)) × 100, and (TN/(TN + FP)) × 100, respectively, are summarized in Table 1. TP, TN, FP, and FN represent True Positive, True Negative, False Positive, and False Negative, respectively. When a single sensor was used separately, the accuracy ranged from 91.8–94.0%, 88.6–90.3%, and 89.8–92.1% for discrimination of geographical origin for garlic, onion, and perilla samples, respectively. For all three cases, use of the radial function with a sigma of 1 and cost constant of 1000 provided the best discrimination accuracy. As highlighted earlier, the responses of each color sensor were individually characteristic and potentially complementary, such that integration of RGB signals obtained from the three color sensors for discrimination would improve accuracy. To test this supposition, the RGB signals of each color sensor were merged, and the merged RGB signals were used for SVM. As shown in Table 1, the accuracy improved to 98.0%, 97.5%, and 97.1% for discrimination of garlic, onion, and perilla samples, respectively. Again, use of the radial function with the same parameters resulted in the most accurate discrimination. 

To statistically assess accuracy improvement, *t*-tests were used. For each sample, the sensor providing the highest accuracy was chosen from among the three sensors (4E sensor for garlic, WHW sensor for onion, WHW sensor for perilla) and the corresponding accuracy was compared with that using the combined Wild + WHW + 4E sensor. The resulting *p*-values for all three cases were much smaller than 0.01 (nearly zero), so accuracy improvement was significant at the 99.0% confidence level. This result demonstrates the utility of combining these three color sensors as a means to enhance sample discrimination.

## 4. Conclusions

Combining the RGB signals obtained from three independent color sensors was effective for improving the discrimination accuracy for garlic, onion, and perilla samples according to their geographical origins. Since M13 bacteriophages composed of different amino acid residues are readily producible by genetic engineering, employing an even larger number of odor-response characteristic color sensors (more than the three used in this study) should provide further improvement of sample discriminability. Therefore, we are currently preparing 3 × 3 and 4 × 4 color sensor arrays to detect and identify hazardous in-house gaseous compounds such as formaldehyde, which causes sick building syndrome [28,29,30,31]. In parallel, an advanced discrimination algorithm able to more effectively handle multi-array RGB data, which are not like conventional visible spectroscopic data, is needed.

## Figures and Tables

**Table 1:** Accuracy, sensitivity, and specificity of discrimination of domestic and imported garlic, onion, and perilla samples using SVM. The numbers in parentheses indicate the corresponding standard deviation in 1000-time cross validation.

Sample	Sensor	Accuracy	Sensitivity	Specificity
Garlic	Wild	91.8% (0.4%)	97.5% (0.5%)	86.1% (0.9%)
	WHW	91.5% (0.5%)	95.3% (0.7%)	87.9% (0.9%)
	4E	94.0% (0.3%)	97.6% (0.4%)	90.4% (0.9%)
	Wild + WHW + 4E	98.0% (0.3%)	98.6% (0.4%)	97.4% (0.4%)
Onion	Wild	89.8% (0.5%)	89.8% (0.9%)	90.0% (1.1%)
	WHW	90.3% (0.5%)	90.3% (1.0%)	90.3% (1.0%)
	4E	88.6% (0.5%)	86.4% (1.1%)	91.0% (1.0%)
	Wild + WHW + 4E	97.5% (0.3%)	97.7% (0.4%)	97.5% (0.5%)
Perilla	Wild	89.8% (0.4%)	86.7% (1.9%)	92.6% (1.6%)
	WHW	92.1% (0.5%)	87.1% (1.0%)	96.9% (0.9%)
	4E	91.6% (0.4%)	94.2% (1.1%)	89.1% (1.2%)
	Wild + WHW + 4E	97.1% (0.4%)	97.4% (1.4%)	96.8% (1.4%)

## Data Availability

Not applicable.
